# Association between diabetes and disease severity in patients with venomous snakebites: A Multicenter Retrospective Analysis

**DOI:** 10.1371/journal.pntd.0012975

**Published:** 2025-04-23

**Authors:** Miaomiao Zhang, Xiuyan Peng, Feng Chen, Qi Li

**Affiliations:** 1 Shengli Clinical Medical College of Fujian Medical University, Fuzhou, Fujian Province, China; 2 Department of Intensive Care Unit, Jiangxi Provincial People’s Hospital, The First Affiliated Hospital of Nanchang Medical College, Nanchang, Jiangxi Province, China; 3 Department of Emergency, Fujian Provincial Hospital, Fuzhou University Affiliated Provincial Hospital, Fujian Provincial Key Laboratory of Emergency Medicine, Fuzhou, Fujian Province, China; Institut de Recherche pour le Développement, FRANCE

## Abstract

**Objective:**

Snakebites remain an overlooked public health issue with high morbidity and mortality. In this study, we assess the impact of diabetes on disease severity in patients with venomous snakebites.

**Methods:**

A retrospective cohort analysis was conducted on snakebite cases treated at eight hospitals in Fujian Province between December 2019 and December 2023. Snakebite severity was evaluated using the Snakebite Severity Score. Univariate and multivariate logistic regression analyses were performed to identify the factors associated with snakebite severity.

**Results:**

The study included 537 patients. The average age of patients is 55 years. 54.93% (n = 295) were aged ≥55 years, 57.17% (n = 307) were male, and 13.41% (n = 72) had diabetes. In the multivariate logistic regression, diabetes (odds ratio [OR] = 5.51; 95% confidence interval [CI]: 3.18–9.55), time from snakebite to hospital (OR = 1.04; 95% CI: 1.01–1.07), and bite site (OR = 0.63; 95% CI: 0.41–0.97) were identified as independent predictors of snakebite severity. Subgroup analysis revealed significant sex differences among patients with diabetes. The odds ratio for moderate-to-severe outcome was 3.81 (95% confidence interval [CI]: 1.81–7.99) in males and 12.57 (95% CI: 5.72–27.60) in females, with an interaction p-value of 0.030. Additionally, diabetes was significantly associated with prolonged hospital length of stay (p < 0.01), increased costs (p < 0.01), higher complication rates (p < 0.01), and a greater likelihood of requiring debridement surgery (p < 0.01) compared to individuals without diabetes.

**Conclusion:**

Diabetes is an independent predictor of disease severity in patients with snakebites, underscoring the need for clinicians to consider the diabetes status when assessing and managing snakebite risk. These findings provide valuable insights for optimizing care strategies for individuals with diabetes who have experienced venomous snakebites.

## Author summary

This study aims to evaluate the impact of diabetes on the severity of venomous snakebites, a significant but often overlooked public health issue. A retrospective cohort analysis was conducted on 537 snakebite patients treated at eight hospitals in Fujian Province between December 2019 and December 2023. The Snakebite Severity Score was used to assess severity, and both univariate and multivariate logistic regression analyses were performed to identify contributing factors. The results showed that diabetes was a significant independent predictor of increased snakebite severity, with an odds ratio (OR) of 5.51. Other factors influencing severity included the time from snakebite to hospital and the bite site. Subgroup analysis revealed notable sex differences, with females with diabetes having a much higher risk of moderate-to-severe outcome than males. Additionally, diabetes was associated with longer hospital stays, higher medical costs, increased complication rates, and a higher likelihood of requiring debridement surgery. These findings highlight diabetes as an important risk factor for worsened outcomes in patients with venomous snakebites, emphasizing the need for more focused care and intervention in this population.

## Introduction

Snakebites are a significant global public health issue that severely impacts morbidity and mortality. Annually, an estimated 81,000–138,000 people die from snakebites worldwide [1]. In China, particularly in the regions south of the Yangtze River, diverse climates and geographical conditions contribute to a high incidence of snakebites, with millions of cases occurring yearly. Among these cases, 100,000–300,000 people require hospitalization for venomous snakebites, with a mortality rate of approximately 5%. These injuries primarily affect the working population in rural and remote areas, resulting in a substantial loss of labor resources [2]. The absence of a comprehensive epidemiological surveillance system in China suggests that the actual incidence of snakebites may be significantly underestimated. To address the global health burden of snakebites, the World Health Organization (WHO) set a target in 2019 to reduce snakebite-related mortality and disability by 50% by 2030 [3].

The effects of snake venom are multifaceted, disrupting normal physiological processes such as neurotransmission and blood coagulation, leading to severe local tissue damage [4]. Complications can include compartment syndrome, necrotizing fasciitis, and gangrene [5]. Each year, approximately 450,000 people worldwide suffer permanent disabilities, such as amputations and limb deformities, as a result of snakebites [4]. To assess the severity of snakebites, Dart et al. developed the Snakebite Severity Score (SSS), which provides an objective tool for evaluating clinical outcomes in patients who experience snakebites [6].

In parallel, diabetes—a chronic metabolic disorder and one of the leading causes of death globally—is on the rise. The International Diabetes Federation projects that the global diabetic population will reach 578 million by 2030 and 700 million by 2045, accounting for 10.9% of the worldwide population [7]. Patients with diabetes often experience immune dysfunction as well as vascular and nerve damage, making them more susceptible to infection and delayed wound healing. Individuals with diabetes face increased challenges in wound healing and prognosis following trauma [8].

Although some studies have explored factors influencing snakebite severity [9–12], limited research exists on the impact of diabetes on snakebite severity. This study aims to examine the relationship between diabetes and snakebite severity by conducting a multicenter retrospective analysis.

## Materials and methods

### Ethics statement

This study adhered to the ethical principles outlined in the Declaration of Helsinki and was approved by the Ethics Committee of Fujian Provincial Hospital (approval number: K2024-09–044). Given the retrospective nature of this study, the requirement for informed consent was waived. All patient data were anonymized before the analysis.

### 1. Study design and participants

This is a multicenter, retrospective study. We retrospectively collected data from 537 venomous snakebite patients treated in eight hospitals in the Fujian province between December 2019 and December 2023. The hospitals included were Fujian Provincial Hospital, Zhangzhou Municipal Hospital of Fujian Province, Pingnan County Hospital, Second Hospital of Licheng District in Putian, Snakebite Prevention and Treatment Hospital of Pucheng County, Second Hospital of Sanming, Tingzhou Hospital of Fujian Province, and Wuyishan Municipal Hospital.

### 2. Inclusion and exclusion criteria

The inclusion criteria were as follows: (1) age ≥ 18 years; (2) Patients diagnosed with venomous snakebites and inpatient for treatment. The exclusion criteria were as follows: (1) Patients with previously undiagnosed diabetes but with elevated blood glucose levels at admission; (2) Re-hospitalizations for the same snakebite incident; (3) Patients with incomplete medical records.

### 3. Observations and statistical indicators

We manually collected patient information using a standardized form from the patient case records of eight hospitals. The collected data included demographic characteristics (sex, age, and occupation), clinical characteristics (bite site, bite location, time from snakebite to hospital, antivenom administration, types of venomous snake, debridement status, hospital length of stay, and hospitalization costs), and laboratory results at admission. The laboratory parameters included white blood cell (WBC) count, neutrophil-to-lymphocyte ratio (NLR) [13], hemoglobin (HB), alanine aminotransferase (ALT), aspartate aminotransferase (AST), blood urea nitrogen (BUN), serum creatinine (SCr), lactate dehydrogenase (LDH), creatine kinase (CK), and creatine kinase isoenzyme MB (CK-MB). The diagnosis of venomous snakebite was established by the treating team based on the patient’s history, physical examination findings (e.g., fang marks and spacing), systemic symptoms, laboratory results, and snake images [14].

### 4. Outcomes

The primary outcome variable was SSS. The severity of snakebite was assessed using the Snakebite Severity Score (SSS) [6], which evaluates six domains (Including the Pulmonary system, Cardiovascular system, Local wound, Gastrointestinal system, Hematologic symptoms, and Central nervous system). The SSS score was retrospectively calculated by the treatment team based on the local or systemic symptoms and the initial laboratory results from the patient’s case records, with scores categorized as mild (0–3 points), moderate (4–7 points), and severe (≥8 points). The secondary outcomes included debridement, complications, length of hospital stay, and costs. Diabetes was one of the independent variables. Patients with diabetes were those who had been diagnosed with diabetes before admission, following the diagnostic criteria outlined in the 2024 American Diabetes Association (ADA) Standards of Medical Care in Diabetes [15].

### 5. Statistical methods

Initial data processing was performed, with cases containing missing data excluded from the analysis. Descriptive statistics were used to summarize patient characteristics. The Shapiro–Wilk test was applied to assess the normality of continuous variables. Normally distributed continuous variables are expressed as mean ± standard deviation, whereas non-normally distributed variables are presented as median and interquartile range (IQR). Categorical variables are reported as counts and percentages.

For comparisons, normally distributed continuous variables were analyzed using the t-test, whereas non-normally distributed continuous variables were compared using nonparametric rank-sum tests. Categorical variables were compared using the chi-square (χ²) or Fisher’s exact test.

Factors influencing snakebite severity were identified through univariate and multivariate logistic regression analyses. Variables showing p < 0.05 in the univariate analysis were included in the multivariate model by the stepwise backward selection method. Pearson’s chi-square test was used for the correlation analysis. Statistical analyses were conducted using R software (version 4.4.0), with p-values < 0.05 considered statistically significant.

## Results

### Demographic and clinical characteristics

**Fig 1:**
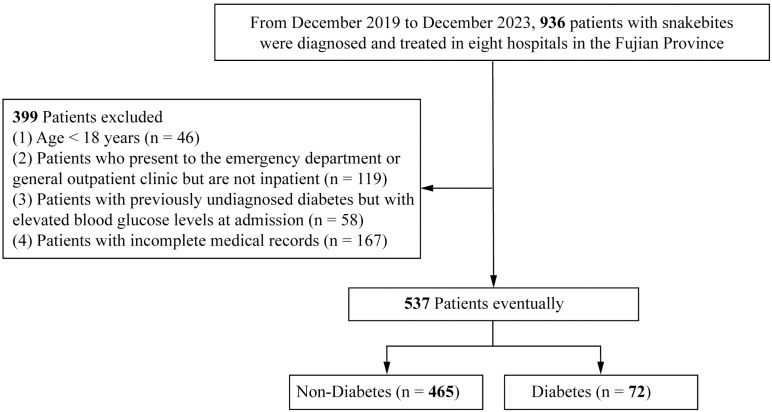
Flow chart of patients.

A total of 537 patients from eight hospitals in Fujian Province were included (Fig 1). All patients presented varying degrees of symptoms of venomous snakebites, such as severe pain, numbness, and swelling. Of these, 465 patients (86.59%) were classified into the non-diabetic group, while 72 patients (13.41%) were categorized into the diabetic group. Detailed demographic and clinical characteristics of both groups are presented in Table 1. Overall, the average age of patients is 55 years. 54.93% (n = 295) of the patients were aged ≥55 years, with a male predominance (57.17%, n = 307). Farmers constituted the majority of the cases (85.10%, n = 457), with most incidents occurring on farmlands (75.05%, n = 403) and primarily affecting the upper limbs (55.12%, n = 296). The most common snake species was categorized as “Others or negative identification” (51.21%, n = 275), followed by “Trimeresurus” (32.77%, n = 176). Additionally, the majority of patients (91.81%, n = 493) were treated with antivenom.

**Table 1:** Baseline characteristics of the patients.

Variables	Total (n = 537)	Non-Diabetes (n = 465)	Diabetes (n = 72)	*p*-value
Age, years^*^	55.00 (48.00–64.00)	55.00 (47.00–63.00)	61.50 (53.00–69.00)	<.001
Age, n (%)				0.004
<55 years	242 (45.07)	221 (47.53)	21 (29.17)	
≥55 years	295 (54.93)	244 (52.47)	51 (70.83)	
Sex, n (%)				0.037
Male	307 (57.17)	274 (58.92)	33 (45.83)	
Female	230 (42.83)	191 (41.08)	39 (54.17)	
Occupation, n (%)				<.001
Farmer	457 (85.10)	408 (87.74)	49 (68.06)	
Non–farmer	80 (14.90)	57 (12.26)	23 (31.94)	
Bite Site, n (%)				0.034
Upper Limb	296 (55.12)	248 (53.33)	48 (66.67)	
Lower limb	241 (44.88)	217 (46.67)	24 (33.33)	
Bite Location, n (%)				0.005
Farmland	403 (75.05)	361 (77.63)	42 (58.33)	
Residential sites	43 (8.01)	33 (7.10)	10 (13.89)	
Factories and highways	31 (5.77)	25 (5.38)	6 (8.33)	
Others	60 (11.17)	46 (9.89)	14 (19.44)	
Time from Snakebite to Hospital, hour^*^	2.00 (1.00–3.00)	2.00 (1.00–3.00)	3.00 (2.00–6.00)	0.015
Types of Venomous Snake, n (%)				<.001
Trimeresurus	176 (32.77)	154 (33.12)	22 (30.56)	
King Cobra	10 (1.86)	4 (0.86)	6 (8.33)	
Deinagkistrodon acutus	70 (13.04)	58 (12.47)	12 (16.67)	
Ovophis monticola	6 (1.12)	4 (0.86)	2 (2.78)	
Others or negative identification	275 (51.21)	245 (52.69)	30 (41.67)	
Severity, n (%)				<.001
Mild	390 (72.63)	364 (78.28)	26 (36.11)	
Moderate-to-severe	147 (27.37)	101 (21.72)	46 (63.89)	
Antivenin, n (%)				0.181
No	44 (8.19)	41 (8.82)	3 (4.17)	
Yes	493 (91.81)	424 (91.18)	69 (95.83)	
WBC, 10^9/L^*^	8.20 (6.47–11.60)	8.10 (6.31–11.28)	9.63 (7.22–13.35)	0.004
HB, g/L^*^	140.00 (129.00–153.00)	140.00 (130.00–153.00)	137.00 (128.50–147.25)	0.123
ALT, U/L^*^	19.00 (14.00–27.00)	19.00 (14.00–27.00)	20.85 (15.00–32.00)	0.056
AST, U/L^*^	23.00 (19.30–29.00)	23.00 (19.30–29.00)	23.00 (19.93–31.15)	0.889
BUN, mmol/L^*^	5.61 (4.64–6.70)	5.66 (4.63–6.77)	5.50 (4.79–6.43)	0.361
SCr, mmol/L^*^	73.00 (59.00–85.00)	72.00 (59.00–85.00)	73.50 (61.10–88.00)	0.588
LDH, U/L^*^	205.00 (177.00–235.00)	203.00 (176.00–234.00)	207.50 (190.00–237.50)	0.343
CK–MB, U/L^*^	18.00 (14.00–23.70)	18.00 (14.00–23.50	16.60 (11.45–24.32)	0.290
CK, U/L^*^	143.00 (100.00–220.00)	143.00 (100.00–220.00)	149.50 (107.75–215.75)	0.619
NLR^*^	4.03 (2.47–7.55)	3.93 (2.31–6.98)	6.09 (3.05–13.10)	<.001

WBC: white blood cell; HB: hemoglobin; ALT: alanine aminotransferase; AST: aspartate aminotransferase; BUN: blood urea nitrogen; SCr: serum creatinine; LDH: lactate dehydrogenase; CK-MB: creatine kinase isoenzyme MB; CK: creatine kinase; NLR: neutrophil-to-lymphocyte ratio; SD: standard deviations.

* Data were expressed as median (interquartile range).

Compared with the non-diabetic group, the diabetic group had a significantly higher proportion of patients with moderate-to-severe cases (63.89% vs. 21.71%, p < 0.001). Moreover, the time from snakebite to hospital admission was longer in the diabetic group [3.00 (2.00–6.00) hours vs. 2.00 (1.00–3.00) hours, p = 0.015]. In addition, the diabetic group demonstrated higher levels of WBC [9.63 (7.22–13.35) 10^9/L vs. 8.10 (6.31–11.28) 10^9/L, p = 0.005] and NLR [6.09 (3.05–13.10) vs. 3.93 (2.31–6.98) p < 0.001] compared to the non-diabetic group, whereas no statistically significant differences were observed for HB, ALT, AST, SCr, LDH, BUN, CK-MB, or CK levels (p > 0.05).

### Factors associated with snakebite severity

Univariate and multivariate analyses were conducted to identify factors associated with snakebite severity (Table 2). In the univariate analysis, factors significantly associated with increased severity included age (p = 0.029), bite site (p = 0.033), bite location (p = 0.004), time from snakebite to hospital (p < 0.001), diabetes (p < 0.001), WBC (p < 0.001), AST (p = 0.047), LDH (p = 0.003), CK-MB (p = 0.012), and NLR (p = 0.004). In the multivariate logistic regression analysis, diabetes (odds ratio [OR] = 5.51; 95% confidence interval [CI]: 3.18–9.55; p < 0.001), time from snakebite to hospital (OR = 1.04; 95% CI: 1.01–1.07; p = 0.008), and bite site (OR = 0.63; 95% CI: 0.41–0.97; p = 0.03) were identified as independent predictors of snakebite severity.

**Table 2:** Uni- and multivariable analyses of snakebite severity.

Variables	Univariable analysis			Multivariable analysis		
OR	95% CI	*p*-value	OR	95% CI	*p*-value
Age						
<55 years	Reference					
≥55 years	1.54	1.04–2.27	0.029			
Sex						
Male	Reference					
Female	0.76	0.52–1.13	0.174			
Occupation						
Farmer	Reference					
Non–farmer	1.43	0.86–2.38	0.167			
Bite Site						
Upper Limb	Reference					
Lower limb	0.66	0.44–0.97	0.033	0.63	0.41–0.97	0.037
Bite Location						
Farmland	Reference					
Residential sites	1.03	0.50–2.11	0.940			
Factories and highways	1.22	0.55–2.74	0.625			
Others	2.29	1.31–4.00	0.004			
Time from Snakebite to Hospital, hour	1.06	1.03–1.09	<.001	1.04	1.01–1.07	0.008
Types of Venomous Snake						
Trimeresurus	Reference					
King Cobra	0.67	0.14–3.25	0.616			
Deinagkistrodon acutus	1.58	0.88–2.84	0.129			
Ovophis monticola	1.58	0.24–7.52	0.744			
Others or negative identification	0.89	0.58–1.37	0.606			
Diabetes						
No	Reference					
Yes	6.38	3.76–10.82	<.001	5.51	3.18–9.55	<.001
WBC, 10^9/L	1.08	1.03–1.13	<.001	1.05	1.00–1.10	0.075
HB, g/L	1.00	0.99–1.01	0.975			
ALT, U/L	1.00	1.00–1.01	0.395			
AST, U/L	1.01	1.01–1.03	0.047			
BUN, mmol/L	0.98	0.92–1.05	0.584			
SCr, mmol/L	1.01	1.00–1.01	0.101			
LDH, U/L	1.01	1.01–1.01	0.003	1.00	1.00–1.01	0.220
CK MB, U/L	1.02	1.01–1.03	0.012	1.01	1.00–1.03	0.119
CK, U/L	1.00	1.00–1.00	0.127			
NLR	1.04	1.01–1.07	0.004			

WBC: white blood cell; HB: hemoglobin; ALT: alanine aminotransferase; AST: aspartate aminotransferase; BUN: blood urea nitrogen; SCr: serum creatinine; LDH: lactate dehydrogenase; CK-MB: creatine kinase isoenzyme MB; CK: creatine kinase; NLR: neutrophil-to-lymphocyte ratio; OR: odds ratio; 95%CI: 95% Confidence Interval.

**Fig 2:**
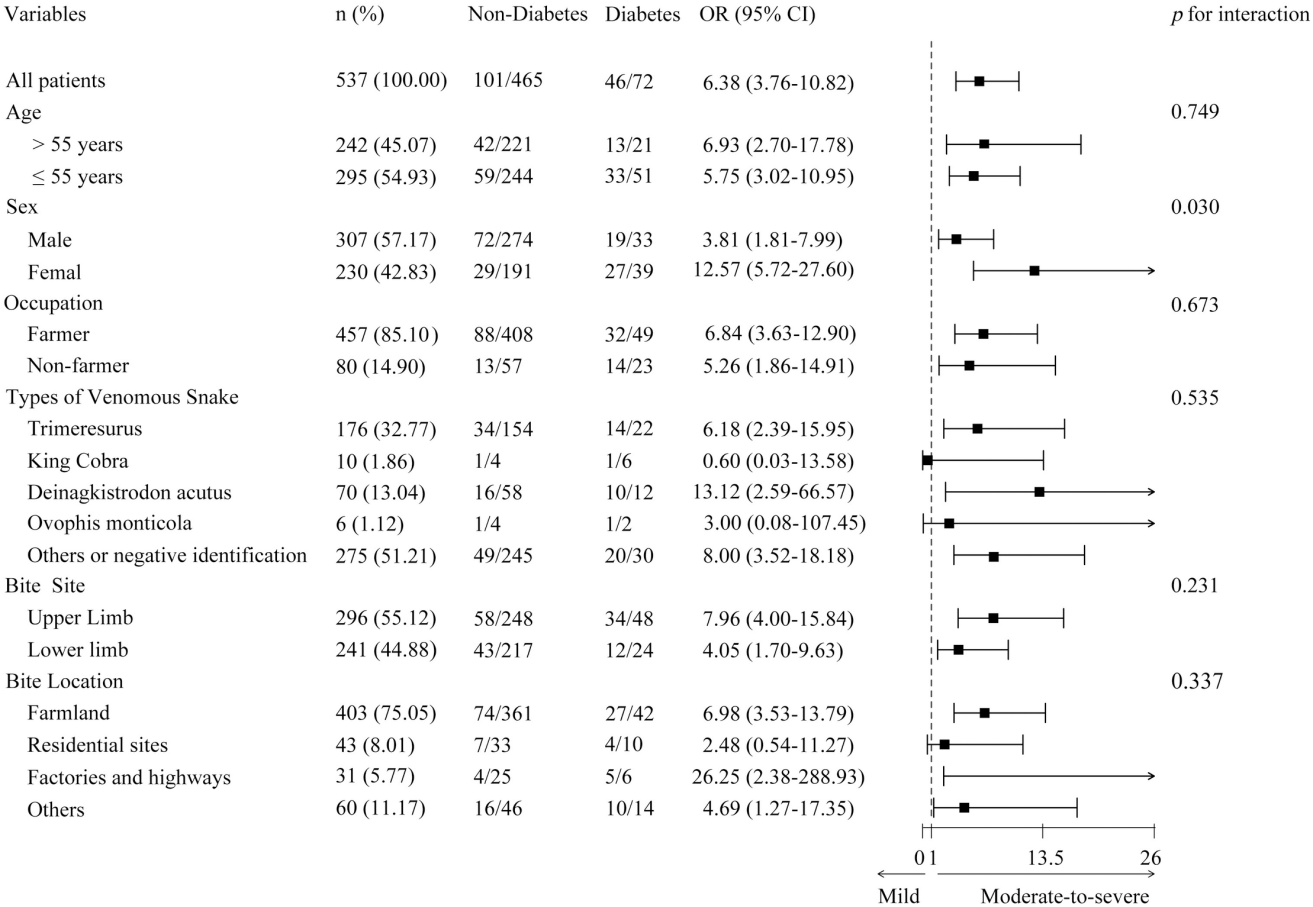
The association between diabetes and snakebite severity across the different subgroups.

The subgroup analysis presented in the forest plot further elucidates the association between diabetes and snakebite severity across the different subgroups (Fig 2). The overall OR for moderate-to-severe snakebite severity in patients with diabetes was 6.38 (95% CI: 3.76–10.82), indicating a strong association between diabetes and increased snakebite severity. Sex was a significant moderating factor in this relationship, with an OR of 3.81 (95% CI: 1.81–7.99) for male patients and 12.57 (95% CI: 5.72–27.60) for female patients, with an interaction p-value of 0.030. This suggests that diabetes had a significantly greater impact on snakebite severity in females than in males.

### Differences in debridement, complications, costs, and hospital stay between snakebite patients with and without diabetes

As shown in Table 3, patients with diabetes experienced significantly longer hospital stays, higher medical costs, increased complication rates, and a higher likelihood of requiring debridement surgery than patients without diabetes. The average hospital length of stay was 5.00 days for patients with diabetes versus 3.00 days for patients without diabetes (p < 0.001). Hospitalization costs were significantly higher for patients with diabetes, with an average of 697.43 USD compared with 483.47 USD for those without diabetes (p < 0.001). Additionally, the probability of debridement surgery was markedly higher in patients with diabetes than in those without diabetes (48.61% vs. 12.04%, p < 0.001), and the complication rate was also higher (37.50% vs. 18.06%; p < 0.001). The S1 Table provides the stratified analysis results that address potential confounding factors, including patient age and the delay between snakebite and hospital admission.

**Table 3:** Differences in debridement, complications, costs, and hospital stay between snakebite patients with and without diabetes.

Variables	Total (n = 537)	Non-Diabetes (n = 465)	Diabetes (n = 72)	*p-*value
Debridement, n (%)				<.001
No	446 (83.05)	409 (87.96)	37 (51.39)	
Yes	91 (16.95)	56 (12.04)	35 (48.61)	
Complication, n (%)				<.001
No	426 (79.33)	381 (81.94)	45 (62.50)	
Yes	111 (20.67)	84 (18.06)	27 (37.50)	
Cost, USD^*^	495.82 (393.68–655.84)	483.47 (383.34–618.86)	697.43 (512.49–1,090.16)	<.001
Hospital length of stay, day^*^	3.00 (2.00–6.00)	3.00 (2.00–5.00)	5.00 (3.00–8.25)	<.001

USD: United States dollar.

* Data were expressed as median (interquartile range).

## Discussion

In this retrospective study, we investigated the association between diabetes and disease severity in patients with venomous snakebites. Multivariate regression analysis revealed that diabetic patients had a significantly higher risk of developing moderate to severe outcome compared to non-diabetic patients (OR = 5.51, p < 0.001). Furthermore, additional analyses showed that diabetic patients exhibited longer hospital stays, higher medical costs, increased rates of complications, and more frequent debridement procedures. Therefore, individualized treatment plans for these patients are warranted, with proactive measures to minimize complications and improve outcomes. Such strategies align with the WHO’s goal of halving snakebite-related morbidity and mortality by 2030. To our knowledge, this is the first study to establish a link between diabetes and worsened clinical outcomes following venomous snakebites.

This study found that venomous snakebites predominantly occurred in males, with most victims being farmers, and the bites primarily taking place in farmland. These findings are consistent with a nationwide random sampling study in China that analyzed risk factors for venomous snakebites. This pattern may be attributed to the overlap between the active periods of venomous snakes—typically early morning or evening in grassy or wooded areas—and the working hours and locations of farmers. Additionally, males constitute the primary workforce in rural areas, further explaining their higher risk of snakebites [2]. Moreover, males constitute the primary workforce in rural regions, further contributing to the high incidence in this demographic. Apart from cases categorized as “others or negative identification”, Trimeresurus bites were the most common, which aligns with epidemiological data from Fujian Province, where Trimeresurus is the predominant venomous snake. Trimeresurus typically hatches in mid-to-late August, and as an ovoviviparous species, their eggs are less susceptible to predation, contributing to their population abundance [16]. Furthermore, we observed that 8.19% of patients did not receive antivenom treatment. In the centers involved in this study, antivenom was not provided free of charge, and most of the venomous snakebite cases involved farmers; therefore, inability to pay may have been one of the main reasons. According to a survey by Hao et al., the reasons for not administering antivenom in China include high costs, lack of availability in healthcare facilities, and patients’ refusal due to insufficient awareness [2]. Additionally, ‘dry bites’ were also one of the reasons why antivenom was not administered clinically [17,18].

We analyzed data from 537 patients, including 72 patients with diabetes (13.41%), aligning with the 12.8% diabetes prevalence rate in China [7]. Multivariate analysis confirmed that diabetes was an independent predictor of snakebite severity; however, the underlying mechanisms remain unclear. Patients with diabetes often exhibit chronic hyperglycemia, metabolic dysfunction, and immune impairment [19], which can lead to a dysregulated wound microenvironment characterized by chronic inflammation, impaired angiogenesis, oxidative stress, and neuropathy [8]. Additionally, immune dysfunction, such as reduced neutrophil and phagocytic activity, compromises the defense of patients with diabetes against toxins [20]. These impairments may contribute to inadequate immune responses to snake venom, potentially exacerbating local tissue damage and systemic toxicity [21,22].

The inflammatory responses induced by snake venom add complexity to these conditions. Snake venom is a potent mixture of enzymes and proteins that disrupt coagulation, induce tissue necrosis, and trigger robust inflammatory responses [4]. Diabetic wounds are prone to prolonged inflammation due to elevated pro-inflammatory mediators and reduced anti-inflammatory cytokines from regulatory cells such as M2 macrophages and regulatory T cells, as demonstrated by Holl et al. [8]. This pro-inflammatory environment, combined with impaired immune function, may exacerbate the impact of venom in patients with diabetes, leading to worse clinical outcomes.

Our subgroup analysis revealed that diabetes significantly increased the risk of severe outcomes across all the subgroups, with a particularly pronounced effect in females. The OR for severe outcomes was 3.81 (95% CI: 1.81–7.99) in males and 12.57 (95% CI: 5.72–27.60) in females, with a significant interaction (p = 0.030). Differences in fat distribution, hormonal profiles, drug metabolism, and adherence between the sexes may account for this disparity. Female patients with diabetes may also have a higher risk of microvascular complications [23,24]. which, combined with the cardiotoxic effects of snake venom [25], can increase their vulnerability to severe outcomes.

Our findings highlight the critical role of timely treatment. The time from snakebite to hospital was a significant risk factor for severity, which is consistent with previous research [2,26,27]. Moderate-to-severe cases presented after an average of 3.00 hours compared with 2.00 hours in mild cases (p < 0.001). This suggests that each hour of delay in seeking treatment increases the risk of disease severity. Similar findings have been reported in other studies. Alfred et al. found that delayed treatment increased the risk of acute kidney injury in patients with snakebites [28]. Mise et al. confirmed that it heightened the risk of acute renal failure and overall toxicity severity [29]. Rafi et al.‘s study also revealed that early detection and treatment of snake venom are the most fundamental and effective approaches for managing venom-induced consumptive coagulopathy [30]. This may be attributed to the rapid dissemination of venom from the bite site into the surrounding tissues, resulting in localized damage or systemic toxicity, including muscle necrosis, coagulation disorders, and neurotoxicity [5]. If patients delay seeking treatment and do not receive antivenom or other emergency interventions, the venom may continue to damage blood vessels, muscles, and nerve tissues. This exacerbates symptoms and increases the risk of adverse outcomes, potentially leading to permanent damage [31].

In this study, the bite site was an important factor influencing the severity of snakebites. Similarly, some studies have suggested that snakebite severity may be related to the bite site [32]. This study shows that patients bitten on the upper limbs account for a higher proportion of cases and are more likely to develop moderate-to-severe outcome. This may be due to the higher number of male patients included in this study, as males are typically involved in activities such as attempts to catch a snake. In the study by Willis et al., it was similarly found that upper limb bites were more common. Their study population consisted predominantly of males, who accounted for 93% of the subjects [33]. The proximity of the upper limbs to the heart may facilitate the rapid return of venom to the heart through the circulatory system, leading to its swift distribution throughout the body and exacerbating systemic toxicity. This can increase the risk of severe complications such as cardiac arrest, shock, and pulmonary hemorrhage [34]. However, the underlying mechanisms remain unclear and warrant further investigation.

Despite the significance of our findings, this study has several limitations. First, as a retrospective analysis, it is subject to potential information bias, such as any non-pharmacological treatments that may have been attempted before hospitalization were not recorded, which may affect the severity of the patient’s condition. Second, we did not adequately explore the impact of different levels of diabetes control (such as glycated hemoglobin levels and blood glucose control during hospitalization) on the severity of snakebite envenomation, which could be a crucial area for future research. Third, the data sample was primarily collected from multiple hospitals in Fujian Province, which may limit the geographical applicability of our findings. Fourth, the SSS was retrospectively assessed by the treatment team, potentially introducing recall and observer bias. Fifth, it is difficult to compare outcomes between the diabetic and non-diabetic subgroups, given the limited number of studies on diabetes patients with snakebite envenomation. Therefore, larger multicenter prospective studies are necessary to validate the results of this study further.

## Conclusion

This study found that diabetes was significantly associated not only with the severity of venomous snakebites but also with hospitalization duration, medical expenses, debridement surgery rates, and complication incidence. Healthcare institutions should enhance monitoring and intervention efforts for patients with diabetes who sustain snakebites to mitigate post-snakebite risks. Future prospective studies are warranted to validate our conclusions.

## Supporting information

S1 Table Differences in debridement, complications, costs, and hospital stay between snakebite patients with and without diabetes: A stratified analysis by age and time from snakebite to hospital. USD: United States dollar. ^*^Data were expressed as median (interquartile range).
(DOCX)
