# Supplementary material for: Association between diabetes and disease severity in patients with venomous snakebites: A Multicenter Retrospective Analysis
Source: PLoS Negl Trop Dis. 2025 Apr 23;19(4):e0012975. doi: 10.1371/journal.pntd.0012975 (PMC12017508; doi:10.1371/journal.pntd.0012975)
Supplement: S1 Table — USD: United States dollar. *Data were expressed as median (interquartile range). (DOCX) [file pntd.0012975.s001.docx]

**S1 Table:** Differences in Debridement, Complications, Costs, and Hospital Stay Between Snakebite Patients with and Without Diabetes: A Stratified Analysis by Age and Time from Snakebite to Hospital

| Variables | Total (n = 537) | Non-Diabetes (n = 465) | Diabetes (n = 72) | *p-*value |
| --- | --- | --- | --- | --- |
| Age<55 years | | | | |
| Debridement, n (%) |  |  |  | <.001 |
| No | 212 (87.60) | 202 (91.40) | 10 (47.62) |  |
| Yes | 30 (12.40) | 19 (8.60) | 11 (52.38) |  |
| Complication, n (%) |  |  |  | 0.181 |
| No | 194 (80.17) | 180 (81.45) | 14 (66.67) |  |
| Yes | 48 (19.83) | 41 (18.55) | 7 (33.33) |  |
| Cost, USD^*^ | 484.15 (377.02–630.12) | 468.02 (372.33–613.35) | 742.35 (491.60–1122.52) | <.001 |
| Hospital length of stay, day^*^ | 3.00 (2.00–5.00) | 3.00 (2.00–5.00) | 5.00 (4.00–8.00) | <.001 |
| Age ≥55 years | | | | |
| Debridement, n (%) |  |  |  | <.001 |
| No | 234 (79.32) | 207 (84.84) | 27 (52.94) |  |
| Yes | 61 (20.68) | 37 (15.16) | 24 (47.06) |  |
| Complication, n (%) |  |  |  | <.001 |
| No | 232 (78.64) | 201 (82.38) | 31 (60.78) |  |
| Yes | 63 (21.36) | 43 (17.62) | 20 (39.22) |  |
| Cost, USD^*^ | 505.29 (413.49–684.96) | 486.66 (398.50–624.53) | 691.29 (517.58–998.92) | <.001 |
| Hospital length of stay, day^*^ | 3.00 (2.00–6.00) | 3.00 (2.00–5.00) | 5.00 (3.00–9.00) | <.001 |
| Time from Snakebite to Hospital ≤ 2 hours | | | | |
| Debridement, n (%) |  |  |  | <.001 |
| No | 262 (81.88) | 246(86.93) | 16(43.24) |  |
| Yes | 58 (18.12) | 37(13.07) | 21(56.76) |  |
| Complication, n (%) |  |  |  | 0.069 |
| No | 267 (83.44) | 240(84.81) | 27(72.97) |  |
| Yes | 53 (16.56) | 43(15.19) | 10(27.03) |  |
| Cost, USD^*^ | 491.80 (398.77–633.09) | 478.75 (393.47–615.70) | 584.69 (520.11–1031.66) | <.001 |
| Hospital length of stay, day^*^ | 3.00 (2.00–5.00) | 3.00 (2.00–5.00) | 5.00 (3.00–8.00) | <.001 |
| Time from Snakebite to Hospital＞2 hours | | | | |
| Debridement, n (%) |  |  |  | <.001 |
| No | 184 (84.79) | 163 (89.56) | 21 (60.00) |  |
| Yes | 33 (15.21) | 19 (10.44) | 14 (40.00) |  |
| Complication, n (%) |  |  |  | 0.001 |
| No | 159 (73.27) | 141 (77.47) | 18 (51.43) |  |
| Yes | 58 (26.73) | 41 (22.53) | 17 (48.57) |  |
| Cost, USD^*^ | 505.29 (383.52–692.07) | 486.81 (374.94–639.76) | 760.02 (524.15–1110.24) | <.001 |
| Hospital length of stay, day^*^ | 3.00 (2.00–6.00) | 3.00 (2.00–5.00) | 7.00 (4.00–9.50) | <.001 |
| USD: United States dollar.  ^*^Data were expressed as median (interquartile range). | | | | |
